# Analysis of Lenvatinib’s Efficacy against Intermediate-Stage Unresectable Hepatocellular Carcinoma

**DOI:** 10.3390/cancers14205066

**Published:** 2022-10-16

**Authors:** Kei Amioka, Tomokazu Kawaoka, Takahiro Kinami, Shintaro Yamasaki, Masanari Kosaka, Yusuke Johira, Shigeki Yano, Kensuke Naruto, Yuwa Ando, Yasutoshi Fujii, Shinsuke Uchikawa, Atsushi Ono, Masami Yamauchi, Michio Imamura, Yumi Kosaka, Kazuki Ohya, Nami Mori, Shintaro Takaki, Keiji Tsuji, Keiichi Masaki, Yoji Honda, Hirotaka Kouno, Hioshi Kohno, Kei Morio, Takashi Moriya, Noriaki Naeshiro, Michihiro Nonaka, Yasuyuki Aisaka, Takahiro Azakami, Akira Hiramatsu, Hiroshi Aikata, Shiro Oka

**Affiliations:** 1 Department of Gastroenterology and Metabolism, Graduate School of Biomedical and Health Sciences, Hiroshima University, Hiroshima 734-8551, Japan; 2 Department of Gastroenterology, Hiroshima Red Cross Hospital & Atomic-Bomb Survivors Hospital, Hiroshima 730-8619, Japan; 3 Department of Gastroenterology, Hiroshima City Asa Citizens Hospital, Hiroshima 731-0293, Japan; 4 Department of Gastroenterology, National Hospital Organization Kure Medical Center and Chugoku Cancer Center, Hiroshima 737-0023, Japan; 5 Department of Gastroenterology, Chugoku Rosai Hospital, Hiroshima 737-0193, Japan; 6 Department of Gastroenterology, National Hospital Organization Higashihiroshima Medical Center, Hiroshima 739-0041, Japan; 7 Department of Gastroenterology, JA Hiroshima General Hospital, Hiroshima 738-8503, Japan; 8 Department of Gastroenterology, Hiroshima Memorial Hospital, Hiroshima 730-0802, Japan

**Keywords:** hepatocellular carcinoma, lenvatinib, intermediate stage, LEN-TACE sequential therapy, radiological response, overall survival, modified Response Evaluation Criteria in Solid Tumors (mRECIST)

## Abstract

**Simple Summary:**

With the emergence of the concepts of transarterial chemoembolization (TACE) refractoriness and unsuitability and recent advances in systemic therapy, treatment options for intermediate-stage, unresectable hepatocellular carcinoma (u-HCC) are increasing. However, the efficacy of lenvatinib and its combination with TACE in clinical practice has not yet been adequately investigated. This study investigated the efficacy of lenvatinib and its combination with TACE after lenvatinib in patients with intermediate-stage u-HCC who were mainly judged to be TACE-refractory or -unsuitable. The results demonstrated the efficacy of lenvatinib and confirmed that further improvements in prognosis could be obtained with TACE after lenvatinib induction. In the intermediate-stage u-HCC, the results suggest it may be important to consider not only the use of lenvatinib but also its combination with TACE to improve patients’ prognosis further, even if they are deemed TACE-refractory or -unsuitable.

**Abstract:**

Transarterial chemoembolization (TACE) has been the standard treatment for intermediate-stage, unresectable hepatocellular carcinoma (u-HCC). However, with recent advances in systemic therapy and the emergence of the concept of TACE-refractory or -unsuitable, the effectiveness of systemic therapy, as well as TACE, has been demonstrated for patients judged to be TACE-refractory or -unsuitable. In this study, the efficacy of lenvatinib and its combination with TACE after lenvatinib was investigated in 140 patients with intermediate-stage u-HCC treated with lenvatinib mainly because of being judged to be TACE-refractory or -unsuitable. Median overall survival (OS) and progression-free survival (PFS) were 24.4 and 9.0 months, respectively, indicating a good response rate. In multivariate analysis, modified albumin–bilirubin (mALBI) grade and up to seven criteria were identified as independent factors for OS, and mALBI grade and tumor morphology were identified as independent factors for PFS. While 95% of all patients were TACE-refractory or -unsuitable, the further prognosis was prolonged by the combination with TACE after lenvatinib initiation. These findings suggest that systemic therapy should be considered for intermediate-stage u-HCC, even in patients judged to be TACE-refractory or -unsuitable. The use of TACE after the start of systemic therapy may further improve prognosis.

## 1. Introduction

Hepatocellular carcinoma (HCC) is a leading cause of cancer-related deaths around the world, and the prognosis is poor for patients with unresectable HCC (u-HCC) [1,2]. Regarding systemic therapy for u-HCC, in 2009, sorafenib was approved as the first molecular-targeted agent. Since 2018, cabozantinib, lenvatinib, ramucirumab, and regorafenib have been approved, thereby expanding the treatment options in Japan [3,4,5,6,7]. Moreover, accompanying the recent development of immunotherapy, in 2020, atezolizumab plus bevacizumab was approved as the first immune combination therapy for u-HCC [8]. At present, with further advances in systemic therapy, six drug regimens expected to improve outcomes have been approved for u-HCC.

For advanced-stage u-HCC, systemic therapy is recommended, and atezolizumab and bevacizumab are becoming the first-line treatment for u-HCC [9]. On the other hand, for intermediate-stage u-HCC, transarterial chemoembolization (TACE) has been recommended as the standard of care. Still, with recent advances in systemic therapy and the emergence of the concepts of refractoriness to and unsuitability for TACE, the efficacy of systemic therapy is being demonstrated [10,11,12,13]. In the latest Barcelona Clinic Liver Cancer (BCLC) staging system, systemic therapy as opposed to TACE, which has been the standard treatment, is recommended for some conditions (diffuse, infiltrative, and extensive bilobar liver involvement) in patients with intermediate-stage HCC [14]. Outcomes of HCC patients treated with TACE and early, not early or not at all followed by sorafenib (the OPTIMIS trial), a prospective, nonrandomized, observational study comparing continued TACE versus switching to sorafenib in the patients’ refractory to or unsuitable for TACE, showed that switching to sorafenib after TACE failure improved patients’ prognosis [15]. Intermediate-stage u-HCC has the potential for cure, but recurrence is common. Therefore, treatment strategies should be developed with attention to not only cure but also the maintenance of the hepatic reserve. Although systemic therapy is being used more frequently in practice, only a limited number of reports have examined the efficacy of systemic therapy in a large number of patients with intermediate-stage u-HCC and treatment strategies for intermediate-stage u-HCC, including the use of TACE and systemic therapy, have not yet been established.

Therefore, in this study, the efficacy of lenvatinib and its combination with TACE after starting lenvatinib was analyzed mainly in patients with intermediate-stage u-HCC considered to be TACE-refractory or -unsuitable.

## 2. Materials and Methods

### 2.1. Patients

In total, 262 patients who had undergone treatment with lenvatinib for u-HCC at our and affiliated institutions from April 2018 to January 2022 provided consent to participate in this study. Among these patients, those with positive hepatitis B virus infection (HBV) surface antigen were considered to have HBV-induced HCC, those with positive anti-hepatitis C virus infection (HCV) antibody were considered to have HCV-induced HCC, and those with negative HBV surface antigen and HCV antibody were considered to have non-B-non-C viral hepatitis (NBNC)-induced HCC. The hepatic reserve was assessed using the Child–Pugh score and modified albumin–bilirubin (mALBI) grade, which was developed to evaluate patients with conventional albumin–bilirubin grade (ALBI) grade 2 more accurately, and is graded on a four-point scale (ALBI score ≤ −2.60 is grade 1, >−2.60 to ≤−2.27 is grade 2a, >−2.27 to ≤−1.39 is grade 2b, and >−1.39 is grade 3) [16,17]. 

The diagnosis of HCC was based on pathology or radiological features of dynamic computed tomography (CT) or magnetic resonance imaging (MRI), such as early dense staining in the arterial phase followed by a washout pattern in the portal/equilibrium phase. The tumor stage was assessed using the BCLC staging system. In addition, patients with BCLC stage B (intermediate stage) were classified using up to seven criteria (maximum tumor diameter (cm) + number of tumors) as an indicator of tumor volume [18]. The inclusion criteria for this study were as follows: Child–Pugh score 5–7, Eastern Cooperative Oncology Group performance status (ECOG PS) of 0 or 1, no extrahepatic metastases, no vascular invasion, and limited to the intrahepatic region. The exclusion criteria were a tumor diameter < 3 cm and fewer than three tumors. Patients who met the inclusion criteria were considered to be in the intermediate stage. A history of prior systemic therapy was not a concern.

### 2.2. Definition of TACE-Refractory and -Unsuitable

In this study, in accordance with previous reports, TACE-refractory was defined as two or more consecutive episodes of residual contrast effect (>50%) of the treated nodule or an increase in the number of intrahepatic tumors compared with the previous TACE procedure, as determined by CT or MRI at 1–3 months after TACE [10,11].

Similarly, TACE-unsuitable was defined as having one or more of the following characteristics at the time of lenvatinib initiation: up to seven criteria out, ALBI grade 2, or non-simple nodular type as conditions predicted to be refractory or resistant to TACE or to result in decreased hepatic reserve with TACE [12,13].

### 2.3. Lenvatinib Treatment Regimens

Oral lenvatinib was started at doses of 8 and 12 mg/day for patients weighing < 60 and ≥60 kg, respectively. The Common Terminology Criteria for Adverse Events version 5.0 were used to evaluate adverse events. The dose of lenvatinib was reduced as necessary based on current dosing guidelines in cases of drug-related adverse events and discontinued in cases of unacceptable serious adverse events. Treatment with lenvatinib was continued in the patients until death or until meeting one of the following criteria: disease progression after treatment, the occurrence of an adverse event necessitating treatment discontinuation, deterioration of ECOG PS to 4, a decline in hepatic reserve or withdrawal of consent to participate in the present study.

### 2.4. Assessment of Response to Lenvatinib

Assessments of radiological response by dynamic CT or MRI were performed at 4–6 and 8–12 weeks after lenvatinib initiation the first and second times, and then every 4–8 weeks thereafter. To assess treatment response, the Response Evaluation Criteria in Solid Tumors (RECIST) version 1.1 and modified Response Evaluation Criteria in Solid Tumors (mRECIST) guidelines were used [19,20]; these guidelines were also used to evaluate the overall response rate (ORR) and disease control rate (DCR). Objective response (OR) was defined as when a complete response (CR) or partial response (PR) was obtained, and non-OR was defined as stable disease (SD) or progressive disease (PD). Overall survival (OS) was defined as the time from the initiation of lenvatinib to death from any cause, with the last follow-up date as the censoring date for surviving patients. Progression-free survival (PFS) was defined as the time from the initiation of lenvatinib to the time of radiological progression by mRECIST or any cause of death, and for patients who were alive without radiological progression, the date of the last radiological evaluation or the date of the switch to the next treatment was determined as the censoring date. TACE events were not considered censoring dates with regard to PFS.

### 2.5. Lenvatinib-TACE Sequential Therapy

TACE was added after lenvatinib was started, depending on the radiotherapy evaluation. The attending physician decided whether to add TACE to each patient. TACE consisted of intra-arterial injection of lipiodol plus cisplatin or epirubicin, followed by injection of an embolic agent (Gelpart) to interrupt blood flow. Cisplatin was used in principle, and in patients at risk of cisplatin allergy, epirubicin was used. In cases in which the tumor responded to the TACE combination, TACE was repeated as needed. Lenvatinib was withdrawn for at least two days before and after TACE and then resumed at the same dose as before withdrawal after confirming that the patient’s general condition and hepatic reserve were satisfactory.

### 2.6. Statistical Analysis

All statistical analyses were performed using the Kaplan–Meier method, log-rank test, Cox proportional hazards analysis, Chi-squared test, Mann–Whitney *U*-test, and logistic regression analysis. A *p*-value < 0.05 was considered significant. All statistical analyses were carried out using IBM SPSS (v.22.0.0).

## 3. Results

### 3.1. Clinical Characteristics of the Participating Patients

Of the 262 patients who consented to participate in this study, 140 (123 men and 17 women) with BCLC stage B intermediate-stage, hepatic reserve Child–Pugh with a score of 5–7 points, and ECOG PS 0 or 1 were included, whereas 5 in the early stage and 117 in the advanced stage were excluded. Table 1 shows the patients’ background characteristics. The median age was 75 (range, 46–90) years, with 14 cases of HBV, 50 cases of HCV, and 76 cases of NBNC as the cause of HCC. The Child–Pugh score at the initiation of lenvatinib was 5 in 89 patients, 6 in 42, and 7 in 9, and the mALBI grade was 1 in 58 patients, 2a in 34, and 2b in 48. Nine cases had a relative tumor volume of ≥50%, and with respect to up to seven criteria, 48 cases were in, and 92 were out. Regarding the percentage of TACE-refractory or -unsuitable cases, 10 were TACE-refractory, 49 were TACE-unsuitable, 74 were TACE-refractory plus -unsuitable, and 7 met none of the above criteria; therefore, most cases (95%) included in this study were TACE-refractory or -unsuitable. Lenvatinib was initiated in 118 patients as first-line systemic therapy and in 22 as second-line or later (nine were sorafenib to lenvatinib, three were atezolizumab and bevacizumab to lenvatinib, two were investigational drug to lenvatinib, seven were sorafenib to regorafenib to lenvatinib, and one was investigational drug to sorafenib to regorafenib to lenvatinib). In total, 51 patients received TACE during the course of lenvatinib treatment, and the median time from lenvatinib initiation to the first TACE combination was 85 (range, 10–371) days. The median observation period was 17.7 (range, 1.0–45.0) months.

### 3.2. Treatment Response and Survival

**Figure 1:**
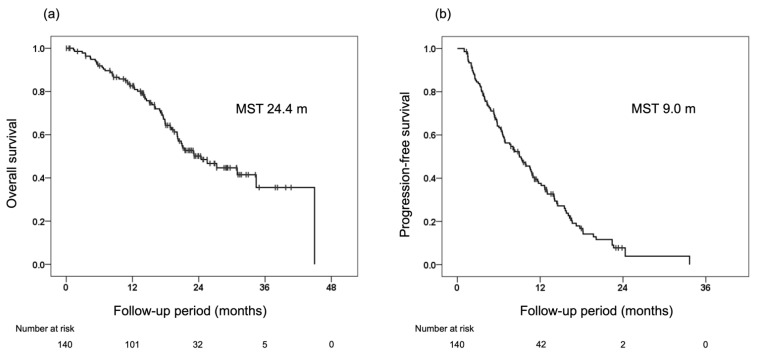
Overall survival (OS) and progression-free survival (PFS) from the initiation of lenvatinib in the 140 patients included in this study. (**a**) OS from the initiation of lenvatinib (median survival time, 24.4 months). (**b**) PFS from the initiation of lenvatinib (median survival time, 9.0 months).

The median OS and PFS of the 140 eligible patients as assessed by mRECIST were 24.4 and 9.0 months, respectively (Figure 1). The median OS/PFS was 23.0/9.0 months for patients with TACE-refractory or -unsuitable, and OS/PFS was not-reached/18.2 months for patients with neither TACE-refractory nor -unsuitable.

The percentages of CR, PR, SD, and PD at the best response assessed by RECIST were 5.0%, 35.0%, 39.3%, and 16.4%, respectively, with an ORR of 40.0% and a DCR of 79.3%. The percentages of CR, PR, SD, and PD at the best response as assessed by mRECIST were 21.4%, 38.6%, 17.1%, and 15.7%, respectively, with an ORR of 60.0% and a DCR of 77.1% (Table 2). In addition, at the first response evaluation, the ORR and DCR were 25.7% and 77.9% for RECIST and 49.3% and 75.0% for mRECIST, respectively; at the second response evaluation, the ORR and DCR were 21.4% and 57.9% for RECIST and 37.1% and 55.7% for mRECIST, respectively. Both RECIST and mRECIST evaluations confirmed a good ORR and DCR at each response evaluation.

### 3.3. OS for Each Initial Radiological Response and Prognostic Factors for OS

**Figure 2:**
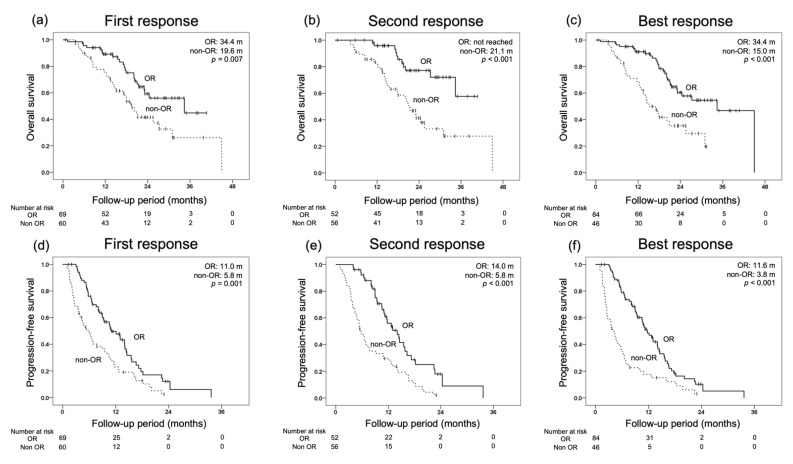
Comparison of overall survival (OS) and progression-free survival (PFS) by the response at the first, second, and best responses as evaluated by modified Response Evaluation Criteria in Solid Tumors (mRECIST). (**a**) OS at the first response (objective response (OR) 34.4 months, non-OR 19.6 months, *p* = 0.007). (**b**) OS at the second response (OR not-reached, non-OR 21.1 months, *p* < 0.001). (**c**) OS at the best response (OR 34.4 months, non-OR 15.0 months, *p* < 0.001). (**d**) PFS at the first response (OR 11.0 months, non-OR 5.8 months, *p* = 0.001). (**e**) PFS at the second response (OR 14.0 months, non-OR 5.8 months, *p* < 0.001). (**f**) PFS at the best response (OR 11.6 months, non-OR 3.8 months, *p* < 0.001).

To confirm that obtaining a radiological response is associated with improved prognosis, OS and PFS were compared between the two groups, OR and non-OR, for the first, second, and best response evaluations for mRECIST, respectively, as a responder analysis (Figure 2). The median OS rates by the first, second, and best response evaluations were 34.4, not-reached, and 34.4 months in the OR group and 19.6, 21.1, and 15.0 months in the non-OR group, respectively. The median PFS rates for mRECIST by the first, second, and best effect measures were 11.0, 14.0, and 11.6 months in the OR group, and 5.8, 5.8, and 3.8 months in the non-OR group, respectively. Significant differences in both OS and PFS were observed between the OR and non-OR groups in the first, second, and best response evaluations.

### 3.4. Prognostic Factors for OS and PFS

Factors contributing to OS and PFS were analyzed in the 140 patients included in this study (Table 3).

On univariate analysis, factors contributing to OS included PS, mALBI grade, up to seven criteria, relative tumor volume, serum alpha-fetoprotein (AFP) value before lenvatinib initiation, tumor morphology, and history of systemic therapy. On multivariate analysis, the following independent factors contributing to OS were identified: mALBI grade (hazard ratio [HR], 1.998; 95% confidence interval [CI], 1.149–3.476; *p* = 0.014) and tumor morphology (HR, 2.105; 95% CI, 1.093–4.052; *p* = 0.026).

Next, on univariate analysis, factors contributing to PFS included mALBI grade, up to seven criteria, and tumor morphology. On multivariate analysis, the following independent factors contributing to PFS were identified: mALBI grade (HR, 1.696; 95% CI, 1.141–2.521; *p* = 0.009) and up to seven criteria (HR, 1.706; 95% CI, 1.080–2.695; *p* = 0.022).

Based on these results, the prognostic analysis was continued, focusing on response at the first radiological evaluation and the combination with TACE, in addition to mALBI grade, up to seven criteria, and tumor morphology, which was shown to be independent factors in the multivariate analysis of OS or PFS.

OS by mALBI grade was 31.0 months in the group with mALBI grade 1–2a and 19.1 months in the group with mALBI grade 2b; by up to seven criteria, 31.0 months in the group with up to seven criteria in and 20.3 months in the group with up to seven criteria out; by tumor morphology, 34.3 months in the group with simple nodular (SN) type and 19.6 months in the group with non-SN type; by the response at first radiological evaluation, 34.4 months for the OR group and 19.6 months for the non-OR group; and by the combination with TACE, 45.0 months in the group with TACE and 20.2 months in the group without TACE.

Next, PFS by mALBI was 10.8 months in the group with mALBI grade 1–2a and 5.8 months in the group with mALBI grade 2b; by up to seven criteria, 12.1 months in the group with up to seven criteria in and 7.8 months in the group with up to seven criteria out; by tumor morphology, 10.7 months in the group with SN type and 7.6 months in the group with non-SN type; by the response at first radiological evaluation, 11.0 months for the OR group and 5.8 months for the non-OR group; and by the combination with TACE, 11.8 months in the group with TACE and 6.5 months in the group without TACE.

### 3.5. Lenvatinib in Combination with TACE

We previously reported the efficacy of lenvatinib–TACE (LEN-TACE) sequential therapy [21]. First, as in our previous report, we performed propensity score matching analysis of patient backgrounds to compare the prognosis of patients who did and did not receive TACE after lenvatinib induction (Tables S1 and S2). The results showed that OS and PFS were stratified between the two groups (Figure S1).

**Figure 3:**
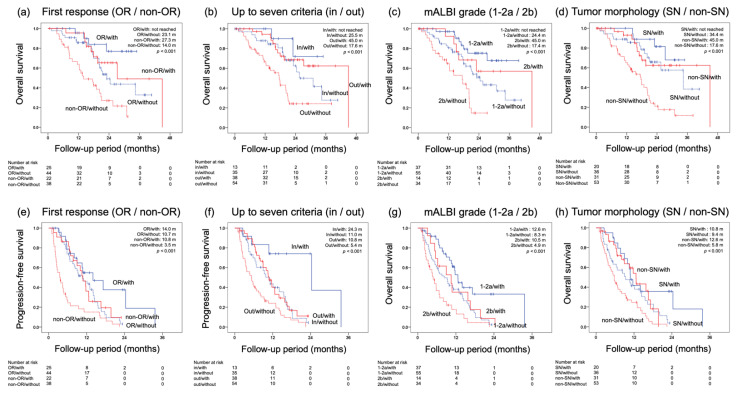
Comparison of overall survival (OS) and progression-free survival (PFS) by four factors, focusing on transarterial chemoembolization (TACE) in combination. (**a**) OS by the response at the first evaluation and TACE combination (objective response (OR)/with TACE not-reached, OR/without TACE 23.1 months, non-OR/with TACE 27.3 months, non-OR/without TACE 14.0 months, *p* < 0.001). (**b**) OS by up to seven criteria and TACE combination (up to seven criteria in/with TACE not-reached, up to seven criteria in/without TACE 25.5 months, up to seven criteria out/with TACE 45.0 months, up to seven criteria out/without TACE 17.6 months, *p* < 0.001). (**c**) OS by tumor morphology and TACE combination (simple nodular [SN]/with TACE not-reached, SN/without TACE 34.4 months, non-SN/with TACE 45.0 months, non-SN/without TACE 17.6 months, *p* < 0.001). (**d**) OS by modified albumin–bilirubin (mALBI) grade and TACE combination (mALBI 1–2a/with TACE not-reached, mALBI 1–2a/without TACE 24.4 months, mALBI 2b/with TACE 45.0 months, mALBI 2b/without TACE 17.4 months, *p* < 0.001). (**e**) PFS by the response at the first evaluation and TACE combination (OR/with TACE 14.0 months, OR/without TACE 10.7 months, non-OR/with TACE 10.8 months, non-OR/without TACE 3.5 months, *p* < 0.001). (**f**) PFS by up to seven criteria and TACE combination (up to seven criteria in/with TACE 24.3 months, up to seven criteria in/without TACE 11.0 months, up to seven criteria out/with TACE 10.8 months, up to seven criteria out/without TACE 5.4 months, *p* < 0.001). (**g**) PFS by mALBI grade and TACE combination (mALBI 1–2a/with TACE 12.6 months, mALBI 1–2a/without TACE 8.3 months, mALBI 2b/with TACE 10.5 months, mALBI 2b/without TACE 4.9 months, *p* < 0.001). (**h**) PFS by tumor morphology and TACE combination (SN/with TACE 10.8 months, SN/without TACE 9.4 months, non-SN/with TACE 12.6 months, non-SN/without TACE 5.8 months, *p* < 0.001).

Next, focusing on lenvatinib with or without TACE in combination, OS and PFS were evaluated for each of four factors: response at the first radiological evaluation; up to seven criteria; mALBI grade; and tumor morphology (Figure 3).

By the response at the first radiological evaluation, OS/PFS was not-reached/14.0 months in the OR group with TACE, 23.1/10.7 months in the OR group without TACE, 27.3/10.8 months in the non-OR group with TACE, and 14.0/3.5 months in the non-OR group without TACE. By up to seven criteria, OS/PFS was not-reached/24.3 months in the up to seven criteria in the group with TACE, 25.5/11.0 months in the up to seven criteria in the group without TACE, 45.0/10.8 months in the up to seven criteria out of the group with TACE, and 17.6/5.4 months in the up to seven criteria out of the group without TACE. By mALBI grade, OS/PFS was not reached/12.6 months in the mALBI grade 1–2a group with TACE, 24.4/8.3 months in the mALBI grade 1–2a group without TACE, 45.0/10.5 months in the mALBI grade 2b group with TACE, and 17.4/4.9 months in the mALBI grade 2b group without TACE. By tumor morphology, OS/PFS was not-reached/10.8 months in the SN-type group with TACE, 34.4/9.4 months in the SN-type group without TACE, 45.0/12.6 months in the non-SN-type group with TACE, and 17.6/5.8 months in the non-SN-type group without TACE. Further significant prognostic stratification was confirmed by the combination with TACE for all four factors.

### 3.6. Conversion Therapy after Initiation of Lenvatinib

**Figure 4:**
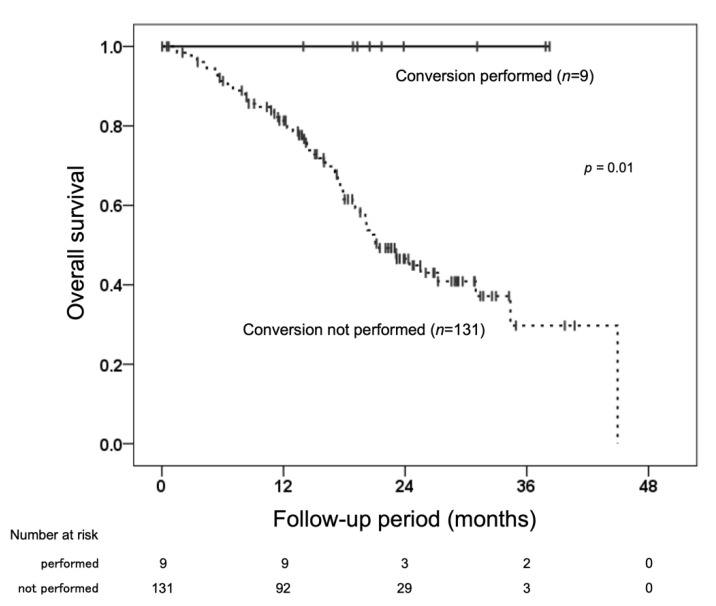
Comparison of overall survival (OS) by conversion therapy.

Of the 140 patients in this study, 9 (6.4%) received lenvatinib followed by local therapy as conversion therapy (Figure 4). This included seven cases of percutaneous radiofrequency ablation, one case of surgical resection, and one case of stereotactic body radiation therapy. The background of the nine patients was as follows: median age 76 (range, 61–88) years; eight men, one woman; mALBI grade 1 in seven patients, 2a in one, and 2b in one; six patients were up to seven criteria in, and three were up to seven criteria out; three had TACE combined; and six had lenvatinib alone. Patients who underwent conversion therapy had higher serum albumin levels and a trend toward a smaller main tumor size, SN type, and lower serum AFP levels. Still, no significant factors were extracted in the regression analysis (Table S3). The median observation period of the nine patients was 21.7 (range, 14.0–38.3) months, and all patients were able to achieve cancer-free and drug-free status.

### 3.7. Subsequent Therapy after Lenvatinib

The details of 119 patients after discontinuation of lenvatinib, excluding 9 who received conversion therapy after starting lenvatinib and 12 who were still on lenvatinib, were as follows: 72 patients switched subsequent systemic therapy (atezolizumab plus bevacizumab in 35, sorafenib in 21, ramucirumab in 11, regorafenib in 2, cabozantinib in 1, and investigational drug in 2), 16 received TACE, and 31 were shifted to best supportive care.

## 4. Discussion

In the past, TACE was recommended as the standard treatment for intermediate-stage u-HCC. However, there have been several reports of cases in which the addition of TACE resulted in difficult tumor control and worsening hepatic reserve, suggesting that switching to systemic therapy rather than TACE may contribute to a better prognosis [22,23,24,25]. Therefore, the concept of TACE refractoriness was defined to facilitate the switch to systemic therapy at the appropriate time [10,11]. Although there is consensus on this concept, many patients continue to receive TACE until they are deemed TACE-refractory, resulting in decreased hepatic reserve and difficulty switching to systemic therapy. 

In recent years, the concept of TACE-unsuitable has been proposed for conditions that are prone to TACE refractoriness, conditions in which hepatic reserve is likely to worsen with TACE, and conditions in which TACE is not expected to be effective [12,13]. As it becomes clear that conditions exist that are refractory to or unsuitable for TACE, and as advances in systemic therapy continue to demonstrate the efficacy of systemic therapy for intermediate-stage u-HCC, a new consensus that systemic therapy is recommended for patients who are refractory to or unsuitable for TACE is emerging. However, only a few reports have examined the efficacy of systemic therapy in intermediate-stage u-HCC in a large number of patients. This study analyzed the prognosis of patients with intermediate-stage u-HCC who received lenvatinib as systemic therapy, mainly including those refractory to or unsuitable for TACE.

The results of the present study confirm the high response rate and prognostic value of lenvatinib for the best response in both RECIST and mRECIST radiological evaluations and demonstrate the efficacy of lenvatinib for intermediate-stage u-HCC in which TACE is the standard treatment (ORR for RECIST, 40.0%; ORR for mRECIST, 60.0%; median OS, 24.4 months; median PFS, 9.0 months). Furthermore, to confirm the efficacy of radiological response with mRECIST, landmark analysis was performed at the first, second, and best response evaluations as responder analysis. The results showed that obtaining a response in intermediate-stage u-HCC could be expected to improve the prognosis of both OS and PFS.

On univariate and multivariate analyses examining factors contributing to OS and PFS, mALBI grade and tumor morphology were identified as independent factors for OS, whereas the mALBI grade and up to seven criteria were identified as independent factors for PFS. In addition, focusing on the combination with TACE, each of the other four factors were evaluated, including the three factors extracted as independent factors (up to seven criteria, mALBI grade, and tumor morphology) plus response at the first radiological evaluation. It was found that the combination with TACE clearly improved the prognosis of patients who had a response (OR) at the first evaluation, had a relatively low tumor burden (up to seven criteria in), a good hepatic reserve (mALBI grade 1–2a), or an SN-type tumor. On the other hand, the combination with TACE showed a certain prognostic value even in nonresponders (non-OR), patients with a relatively high tumor burden (up to seven criteria out), patients with a poor hepatic reserve (mALBI grade 2b), or patients with a non-SN-type tumor. Although many of the patients in this study were cases of induction due to being TACE-refractory or -unsuitable, the results indicate that lenvatinib induction followed by TACE may further improve prognosis. We suggest that even if patients are TACE-refractory or -unsuitable, further prognostic improvements may be obtained with the combination of lenvatinib followed by TACE, even when deemed refractory or unsuitable.

Of the six systemic therapies approved in Japan for u-HCC, lenvatinib has a particularly high response rate. However, in clinical trials, it has become clear that patients are forced to switch to other therapies for various reasons, including side effects, worsening hepatic reserve, and resistance acquisition. Therefore, a treatment strategy that takes these issues into account in comparison with other drugs is desirable. We have previously reported the efficacy of lenvatinib followed by TACE [21], and the efficacy of LEN-TACE sequential therapy has also been reported [26,27,28]. The advantages of LEN-TACE sequential therapy include the acquisition of a deeper response and the possibility of enhancing the therapeutic effect of TACE by normalization of tumor angiogenesis by preceding systemic therapy [29,30]. In addition, a recent prospective clinical trial reported that the combination of sorafenib and TACE resulted in effective outcomes [31]. The present study showed that not only considering systemic therapy but combining it with conventional TACE may lead to better outcomes in intermediate-stage u-HCC. In intermediate-stage u-HCC, where response should be pursued, especially in patients with lower tumor burden or more localized tumors, the combination of TACE with lenvatinib is a very effective therapeutic strategy that may increase the expectation of cure and make more effective use of lenvatinib, which is expected to respond but has certain limitations for the continuation of treatment. The results of the ongoing clinical trial (TACTICS-L) of the combination of lenvatinib and TACE are awaited [32].

In advanced-stage u-HCC, the treatment strategy combined with TACE for intrahepatic local control, as in the present study, is difficult in terms of vascular invasion and the presence of distant metastases. With recent advances in combination immunotherapy, atezolizumab plus bevacizumab combination therapy is becoming established as the first-line systemic therapy unless certain conditions, such as a history of autoimmune disease, are present. 

On the other hand, intermediate-stage u-HCC is known to have a wide range of tumor factors related to tumor volume and the site of intrahepatic tumor; as a result, therapeutic strategies have not yet been established. Several trials of the efficacy of systemic therapy in intermediate-stage u-HCC are currently underway, and it is expected that the efficacy of systemic therapy will be further established. In fact, a certain percentage of patients with intermediate-stage u-HCC achieve downstaging after receiving lenvatinib, especially in combination with TACE, and are then treated with conversion therapy [33]. In the present study of intermediate-stage HCC, conversion therapy was performed in 6.4% of the patients, and the prognosis was clearly favorable for those who were able to progress to conversion therapy. It is always desirable to consider the possibility of conversion therapy, especially with lenvatinib, which has a high response rate. 

Atezolizumab plus bevacizumab is now widely used in clinical practice, and reports, albeit retrospective, of the efficacy of atezolizumab plus bevacizumab are emerging, even in intermediate-stage u-HCC [34]. As shown in the present study, in patients with a high tumor burden, as typified by up to seven criteria out, the combination of TACE may have a certain prognostic value but is expected to be less curative in comparison with patients with a low tumor burden, as typified by up to seven criteria in, in whom the prognostic value is evident. Therefore, one may need to consider treatment options for these patients at the advanced stage, i.e., atezolizumab plus bevacizumab as the first-line drug, rather than aiming at curative treatment. 

As future issues must considered in systemic therapy for u-HCC in the intermediate stage, further research is needed on the efficacy, safety, and cost–benefit ratio of treatment options, including the use of combined immunotherapy, such as atezolizumab plus bevacizumab, which is becoming established as a standard drug therapy.

This study had several limitations, including its retrospective design, small sample size, and short observation period. The decision to use combination therapy with TACE was made by each attending physician, and the timing of the use of TACE after the initiation of lenvatinib was not constant. There may also be selection bias, such as the fact that TACE was used more often in patients who achieved a response. However, the results of the present study suggest that not only the introduction of lenvatinib as a systemic therapy for patients who are refractory or unsuitable for TACE with intermediate-stage u-HCC, but also the combination with TACE, in cases judged to be TACE-refractory or -unsuitable after the introduction of lenvatinib, may further improve the prognosis. These results indicate that the concept of treatment for u-HCC in the intermediate stage is changing significantly.

## 5. Conclusions

Lenvatinib is effective in intermediate-stage u-HCC, even in patients who are TACE-refractory or -unsuitable, and further prognostic benefit can be obtained with TACE after lenvatinib induction. In intermediate-stage u-HCC, it is important to consider the introduction of lenvatinib and the subsequent use of TACE.

## Figures and Tables

**Table 1:** Clinical characteristics at the initiation of lenvatinib (*n* = 140).

Characteristic	Median (Range) or Patients, *n*
Age, range, *y*	75 (46–90)
Sex (male/female), *n*	123/17
Weight (<60/≥60 kg), *n*	58/82
Performance status (0/1), *n*	128/12
Etiology (HBV/HCV/NBNC), *n*	14/50/76
Total bilirubin, range, mg/dL	0.7 (0.3–2.4)
Albumin, range, g/dL	3.8 (2.7–4.8)
Prothrombin activity, range, %	90 (59–129)
Child–Pugh score (5/6/7), *n*	89/42/9
mALBI grade (1/2a/2b), *n*	58/34/48
Size of main tumor, range, mm	26.0 (8.0–130.0)
Number of tumors (2–3/4–6//≥7), *n*	39/45/56
Relative tumor volume (<50/≥50%), *n*	131/9
Tumor morphology (SN type/non-SN type), *n*	56/84
Serum AFP value, range, ng/mL	16.1 (0.9–84001.6)
Serum DCP value, range, mAU/mL	175.5 (13.0–93112.0)
Up to seven criteria (in/out), *n*	48/92
History of systemic treatment (with/without), *n*	22/118
Number of previous TACE procedure beforelenvatinib (0/1-2//≥3), *n*	37/40/63
TACE-refractory or unsuitable(refractory/unsuitable/refractory plus unsuitable/without), *n*	10/49/74/7
Lenvatinib followed by TACE (with/without), *n*	51/89
Observation period, range, months	17.7 (1.0–45.0)

HBV—hepatitis B virus infection; HCV—hepatitis C virus infection; NBNC—non-B-non-C viral hepatitis; mALBI—modified albumin-bilirubin; SN—simple nodular; AFP—alpha-fetoprotein; DCP—des-γ-carboxy prothrombin; TACE—transarterial chemoembolization.

**Table 2:** Radiological response to lenvatinib.

	RECIST % (*n*)			mRECIST % (*n*)		
	Best	1st	2nd	Best	1st	2nd
CR	5.0 (7)	3.6 (5)	2.1 (3)	21.4 (30)	11.4 (16)	13.6 (19)
PR	35.0 (49)	22.1 (31)	19.3 (27)	38.6 (54)	37.9 (53)	23.6 (33)
SD	39.3 (55)	52.1 (73)	36.4 (51)	17.1 (24)	25.7 (36)	18.6 (26)
PD	16.4 (23)	17.9 (25)	22.9 (32)	15.7 (22)	17.1 (24)	21.4 (30)
NE	4.3 (6)	4.3 (6)	19.3 (27)	7.1 (10)	7.9 (11)	22.9 (32)
ORR	40.0 (56)	25.7 (36)	21.4 (30)	60.0 (84)	49.3 (69)	37.1 (52)
DCR	79.3 (111)	77.9 (109)	57.9 (81)	77.1 (108)	75.0 (105)	55.7 (78)

RECIST—Response Evaluation Criteria in Solid Tumors; mRECIST—modified Response Evaluation Criteria in Solid Tumors; CR—complete response; PR—partial response; SD—stable disease; PD—progressive disease; ORR—overall response rate; DCR—disease control rate.

**Table 3:** Univariate and multivariate analyses of prognostic factors for overall and progression-free survival.

Factors for Overall Survival	Univariate *p*-Value	Multivariate		
		HR	95% CI	*p*-Value
Sex (male vs. female)	0.580			
Etiology (NBNC vs. viral)	0.066			
Performance status (0 vs. 1)	0.040	1.652	0.713–3.831	0.242
mALBI grade (1/2a vs. 2b)	0.002	1.998	1.149–3.476	0.014
Up to seven criteria (in vs. out)	0.046	1.220	0.632-2.354	0.553
Relative tumor volume (<50% vs. ≥50%)	0.045	1.026	0.396–2.653	0.958
Serum AFP value (<400 vs. ≥400), ng/mL	0.025	1.751	0.933–3.286	0.081
Tumor morphology (SN type vs. non-SN type)	<0.001	2.105	1.093-4.052	0.026
History of systemic treatment (without vs. with)	0.038	1.395	0.734–2.652	0.309
TACE-refractory or unsuitable (with vs. without)	0.244			
**Factors for Progression-Free Survival**	**Univariate *p*-Value**	**Multivariate**		
		**HR**	**95% CI**	***p*-Value**
Sex (male vs. female)	0.235			
Etiology (NBNC vs. viral)	0.992			
Performance status (0 vs. 1)	0.063			
mALBI grade (1/2a vs. 2b)	0.003	1.696	1.141–2.521	0.009
Up to seven criteria (in vs. out)	0.006	1.706	1.080–2.695	0.022
Relative tumor volume (<50% vs. ≥50%)	0.343			
Serum AFP value (<400 vs. ≥400), ng/mL	0.933			
Tumor morphology (SN type vs. non-SN type)	0.025	1.270	0.825–1.957	0.277
History of systemic treatment (without vs. with)	0.108			
TACE-refractory or unsuitable (with vs. without)	0.050			

NBNC—non-B-non-C viral hepatitis; mALBI—modified albumin-bilirubin; AFP—alpha-fetoprotein; SN—simple nodular; TACE—transarterial chemoembolization.

## Data Availability

The data that support the findings of this study are available from the corresponding author upon reasonable request.

## Supplementary Materials

The following supporting information can be downloaded at: https://www.mdpi.com/article/10.3390/cancers14205066/s1, Figure S1: Comparison of overall survival (OS) and progression-free survival (PFS) between groups with and without transarterial chemoembolization (TACE) after propensity score matching; Table S1: Clinical characteristics at the initiation of lenvatinib (before propensity score matching); Table S2: Clinical characteristics at the initiation of lenvatinib (after propensity score matching); Table S3: Comparison of clinical characteristics of patients who received conversion therapy and those who did not.
