# Supplementary material for: Analysis of Lenvatinib’s Efficacy against Intermediate-Stage Unresectable Hepatocellular Carcinoma
Source: Cancers (Basel). 2022 Oct 16;14(20):5066. doi: 10.3390/cancers14205066 (PMC9600304; doi:10.3390/cancers14205066)
Supplement: Supplementary file 1 [file cancers-14-05066-s001.zip › cancers-1872897-supplementary.pdf]

Supplementary Material: Analysis of Lenvatinib's Efficacy against Intermediate-Stage Unresectable Hepatocellular Carcinoma

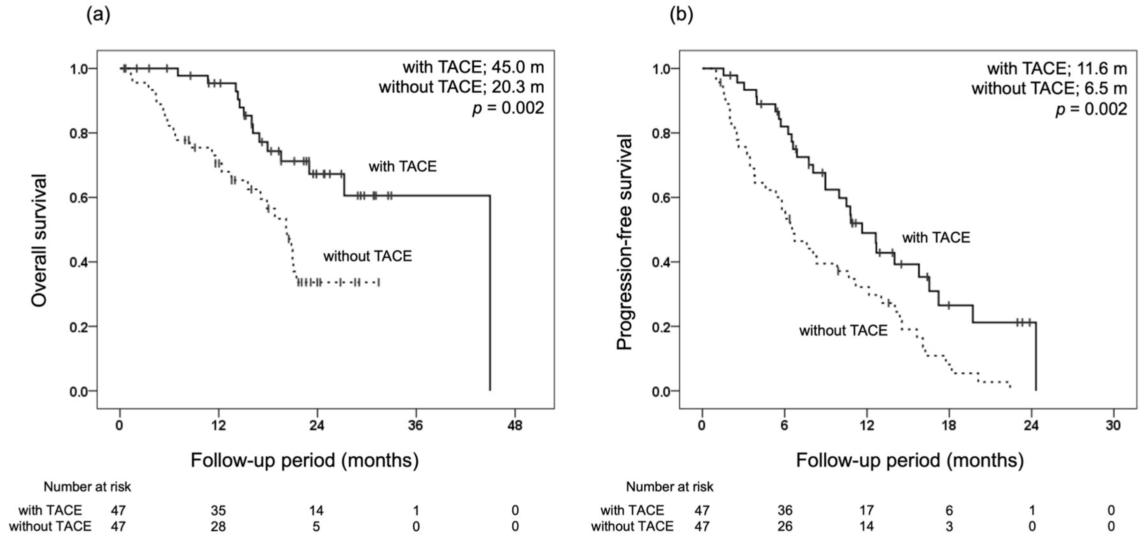

Figure S1. Comparison of overall survival (OS) and progression-free survival (PFS) between groups with and without transarterial chemoembolization (TACE) after propensity score matching. (a) OS in the group with and without TACE (with TACE 45.0 months, without TACE 20.3 months, p = 0.002). (b) PFS in the group with and without TACE (with TACE 11.6 months, without TACE 6.5 months, p = 0.002).

Table S1. Clinical characteristics at the initiation of lenvatinib (before propensity score matching).

Characteristic	With TACE *n* = 51	Without TACE *n* = 89	*p*-Value
Age, range, y	72 (46–90)	77 (48–89)	0.010
Sex (male/female), n	48/3	75/14	0.086
Weight (< 60/ ≥ 60 kg), n	19/32	39/50	0.448
Performance status (0/1), n	48/3	80/9	0.536
Etiology (viral /non-viral), n	22/29	42/47	0.643
Total bilirubin, range, mg/dL	0.7 (0.3–1.7)	0.7 (0.3–2.4)	0.730
Albumin, range, g/dL	3.8 (3.1–4.8)	3.7 (2.7–4.6)	0.207
Prothrombin activity, range, %	92 (61–126)	88 (59–129)	0.330
Child–Pugh score (5-6/7), n	47/4	84/5	0.724
mALBI grade (1-2a/2b), n	37/14	55/34	0.197
Size of main tumor, range, mm	33.0 (10.0–120.0)	23.0 (8.0–130.0)	0.047
Number of tumors (2-3/ > 4), n	12/39	27/62	0.387
Relative tumor volume (< 50/ ≥ 50%), n	47/4	84/5	0.724
Tumor morphology (SN type/non-SN type), n	20/31	36/53	0.886
Serum AFP value, range, ng/mL	12.2 (1.4–56744.0)	17.2 (0.9–84001.6)	0.697
Serum DCP value, range, mAU/mL	241 (13–93112)	151 (14–75549)	0.534
Up to 7 criteria (in/out), n	13/38	35/54	0.097
History of systemic treatment (with/without), n	5/46	17/72	0.146
Number of previous TACE procedure before lenvatinib (0-2/ < 3), n	31/20	46/43	0.298
TACE-refractory or unsuitable (with/without), n	50/1	83/6	0.422

mALBI, modified albumin-bilirubin; SN, simple nodular; AFP, alpha-fetoprotein; DCP, des-γ-carboxy prothrombin; TACE, transarterial chemoembolization.

Table S2. Clinical characteristics at the initiation of lenvatinib (after propensity score matching).

Characteristic	With TACE *n* = 47	Without TACE *n* = 47	*p*-Value
Age, range, y	73 (54–90)	75 (57–89)	0.751
Sex (male/female), n	44/3	43/4	1.000
Weight (< 60/ ≥ 60 kg), n	18/29	16/31	0.668
Performance status (0/1), n	44/3	42/5	0.714
Etiology (viral /non-viral), n	21/26	22/25	0.836
Total bilirubin, range, mg/dL	0.7 (0.3–1.7)	0.8 (0.4–2.4)	0.298
Albumin, range, g/dL	3.8 (3.1–4.7)	3.7 (2.7–4.6)	0.345
Prothrombin activity, range, %	92 (61–114)	88 (65–129)	0.997
Child–Pugh score (5-6/7), n	43/4	44/3	1.000
mALBI grade (1-2a/2b), n	33/14	28/19	0.280
Size of main tumor, range, mm	33.0 (10.0–120.0)	28.0 (10.0–130.0)	0.517
Number of tumors (2-3/ > 4), n	12/35	10/37	0.626
Relative tumor volume (< 50/ ≥ 50%), n	43/4	42/5	1.000
Tumor morphology (SN type/non-SN type), n	19/28	16/31	0.522
Serum AFP value, range, ng/mL	17.0 (1.4–56744.0)	12.2 (2.0–28047.0)	0.809
Serum DCP value, range, mAU/mL	241 (13–93112)	138 (14–60331)	0.615
Up to 7 criteria (in/out), n	12/35	11/36	0.810
History of systemic treatment (with/without), n	5/42	9/38	0.247
Number of previous TACE procedure before lenvatinib (0-2/ < 3), n	29/18	26/21	0.530
TACE-refractory or unsuitable (with/without), n	46/1	45/2	1.000

mALBI, modified albumin-bilirubin; SN, simple nodular; AFP, alpha-fetoprotein; DCP, des-γ-carboxy prothrombin; TACE, transarterial chemoembolization.

Table S3. Comparison of clinical characteristics of patients who received conversion therapy and those who did not.

Characteristic	conversion not performed *n* = 131	conversion performed *n* = 9	Univariate *p*-Value	Multivariate *p*-Value
Age, range, y	75 (46–90)	76 (61–88)	0.444	
Sex (male/female), n	115/16	8/1	0.922	
Weight (< 60/ ≥ 60 kg), n	52/79	6/3	0.112	
Performance status (0/1), n	48/3	9/0	0.342	
Etiology (viral /non-viral), n	62/69	2/7	0.144	
Total bilirubin, range, mg/dL	0.7 (0.3–2.4)	0.7 (0.4–1.0)	0.234	
Albumin, range, g/dL	3.8 (2.7–4.8)	4.1 (3.5–4.6)	0.046	0.068
Prothrombin activity, range, %	90 (59–129)	93 (82–102)	0.277	
Child–Pugh score (5-6/7), n	122/9	9/0	0.416	
mALBI grade (1-2a/2b), n	84/47	8/1	0.130	
Size of main tumor, range, mm	27.0 (8.0–130.0)	12.0 (10.0–120.0)	0.050	0.448
Number of tumors (2-3/ > 4), n	35/96	4/5	0.251	
Relative tumor volume (< 50/ ≥ 50%), n	123/8	9/0	0.445	
Tumor morphology (SN type/non-SN type), n	50/81	6/3	0.091	0.184
Serum AFP value, range, ng/mL	17.4 (1.4–84001.6)	8.9 (0.9–1091.7)	0.097	0.446
Serum DCP value, range, mAU/mL	188 (13–93112)	33 (18–20671)	0.101	
Up to 7 criteria (in/out), n	43/88	5/4	0.165	
History of systemic treatment (with/without), n	22/109	0/9	0.181	
Number of previous TACE procedure before lenvatinib (0-2/ < 3), n	71/60	6/3	0.467	
TACE-refractory or unsuitable (with/without), n	125/6	8/1	0.385	

mALBI, modified albumin-bilirubin; SN, simple nodular; AFP, alpha-fetoprotein; DCP, des-γ-carboxy prothrombin; TACE, transarterial chemoembolization.
